# Chemosensor properties of 7-hydroxycoumarin substituted cyclotriphosphazenes *Dedicated to our supervisor Prof. Dr. Adem Kılıç on his retirement.


**DOI:** 10.3906/kim-1908-51

**Published:** 2020-02-11

**Authors:** Gönül YENİLMEZ ÇİFTÇİ, Sergen YILMAZ, Nagihan BAYIK, Elif ŞENKUYTU, Esra Nur KAYA, Mahmut DURMUŞ, Mustafa BULUT

**Affiliations:** 1 Department of Chemistry, Faculty of Science, Gebze Technical University, Gebze, Kocaeli Turkey; 2 Department of Chemistry, Faculty of Art and Science, Marmara University, Kadıköy, İstanbul Turkey

**Keywords:** Cyclotriphosphazene, coumarin, fluorescent chemosensor, aqueous media

## Abstract

The newly synthesized cyclotriphosphazene cored coumarin chemosensors 5, 6, and 7 were successfully characterized by ^1^ H NMR, ^31^ P NMR, and MALDI-TOF mass spectrometry. Additionally, the photophysical and metal sensing properties of the targeted compounds were determined by fluorescence spectroscopy in the presence of various metals (Li ^+^ , Na ^+^ , K ^+^ , Cs ^+^ , Mg ^2+^ , Ca ^2+^ , Ba ^2+^ , Cr ^3+^ , Mn ^2+^ , Fe ^3+^ , Co ^2+^ , Al ^3+^ , Hg ^+^ , Cu ^2+^ , Zn ^2+^ , Ag ^+^ , and Cd ^2+^ ) . The fluorescence titration results showed that compounds 5, 6, and 7 could be employed as fluorescent chemosensors for Fe ^3+^ ions with high sensitivity. The complex stoichiometry between final cyclotriphosphazene chemosensors and Fe ^3+^ ions was also determined by Job’s plots.

## 1. Introduction

Fluorescence chemosensor design is the key topic in the determination of ions in biological processes. It is important to examine the fluorescence properties of the compounds to be synthesized as new sensors, their location in living organisms, and their usability for metal ion detection as fluorescent sensors [1–3]. Fe ^3+^ , an indispensable element for life, both provides oxygen-forming capacity and serves as a cofactor in many enzymatic reactions in the mitochondrial respiratory chain, and both deficiency and excess amounts of Fe ^3+^ can cause various diseases [4]. Deficiency of Fe ^3+^ ions causes anemia, diabetes, liver damage, Parkinson disease, and cancer. On the other hand, excessive intake of iron in humans is equally harmful and may cause dysfunctions of certain organs, such as the heart, pancreas, and liver [5]. Furthermore, high levels of Fe ^3+^ ions may be toxic, promoting the oxidation of lipids, proteins, and other cellular components. For this reason, the detection of iron ions in biological media is very important [6]. Coumarin-based fluorescent chemosensors show less toxicity and are easily altered. Furthermore, the carbonyl group of coumarin can partake in coordination with metal ions if necessary. This is an ideal model for the design of chemosensors because the response will be fast and efficient when guests, e.g., protons and metal ions, are bound to the host probes. Recently, coumarins being are used as fluorescent chemosensors due to their high optical activities, high light stability, high quantum yield, wide Stokes shift, and low toxicity properties. These compounds are an important part of organic heterocyclic compounds used in sensor technology [4]. Coumarin compounds demonstrate perfect photophysical properties. For this reason, they are used in many applications for their anticoagulant [7], antitumor [8], photosensitizer [9], antiviral [10], pesticidal [11] , antitubercular [12], anticancer [13], anti-HIV [14], and antifungal properties [15]. The fluorescence characteristics of these compounds can be varied by substituting different groups at the 7-position of the coumarin skeleton. Because of its donor–acceptor behavior, it has very high potential to be used as a fluorescent chemosensor [16–18].

Phosphazenes are composed of repeating units of [–P=N–] in their structure. They also have a cyclic structure connecting two inorganic or organic side groups (R) to each phosphorus atom. Hexachlorocyclotriphosphazene is one of the most important members of the phosphazene family because different products are obtained as a result of various reactions on the six chlorine groups in the structure. Depending on the attached groups, they are suitable for the synthesis of different compounds for various applications [19,20]. During the past two decades, nucleophilic substitution reactions at phosphorus atoms of hexachlorocyclotriphosphazene have been extensively explored, leading to an enormous variety of materials with interesting properties such as anticancer [21] and antimicrobial agents [22] or fluorescence sensors [23]. Although there are many studies about coumarin, there are few studies about cyclotriphosphazenes bearing coumarin groups. Some of these were synthesis and characterization studies [24], as well as fluorescent chemosensor studies [25,26]. In the current study, new coumarin-based cyclotriphosphazenes (5–7) (Scheme), which are fluorescent chemosensors for Fe ^3+^ ions, have been successfully designed and prepared. The photophysical properties and metal-sensing behaviors of the new compounds (5–7) were investigated by UV-Vis electronic absorption and florescence spectroscopies.

**Scheme:**
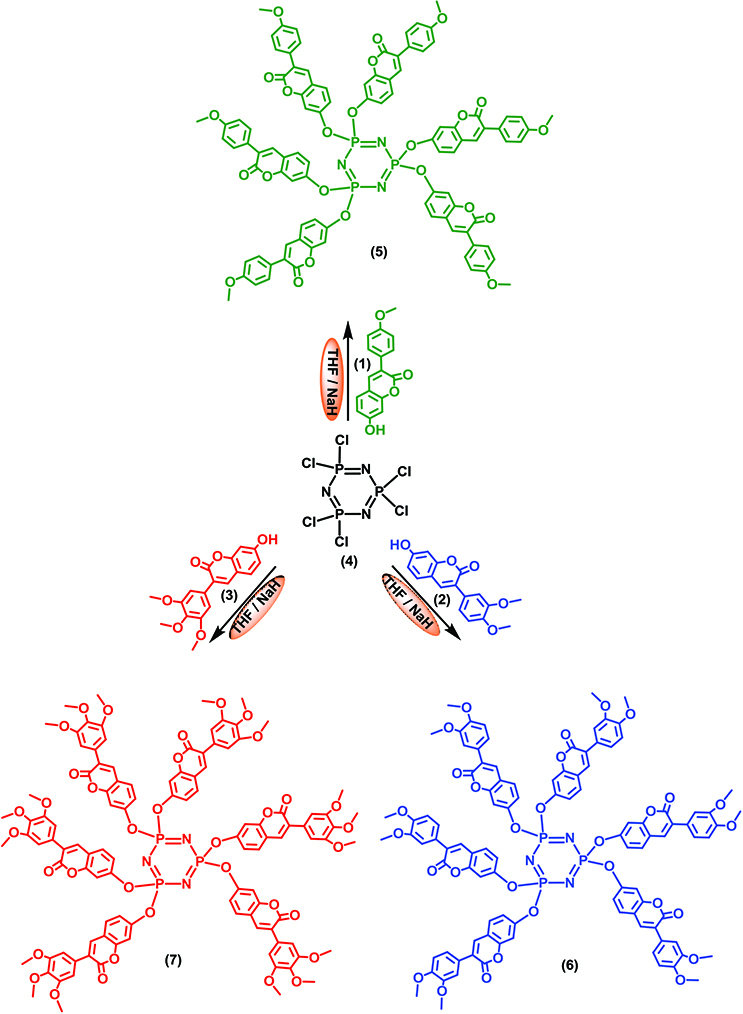
7-Hydroxycoumarin substituted cyclotriphosphazenes (5–7).

## 2. Experimental

Materials and methods are given in the Supporting information.

### 2.1. Syntheses

7-Hydroxy-3-(4-methoxyphenyl)coumarin (1) [27], 7-hydroxy-3-(3,4-dimethoxyphenyl)coumarin (2) [28], and 7- hydroxy-3-(3,4,5-trimethoxyphenyl)coumarin (3) [29] were synthesized according to the procedures given in the literature.

#### 2.1.1. Synthesis of compound 5

**Figure 1:**
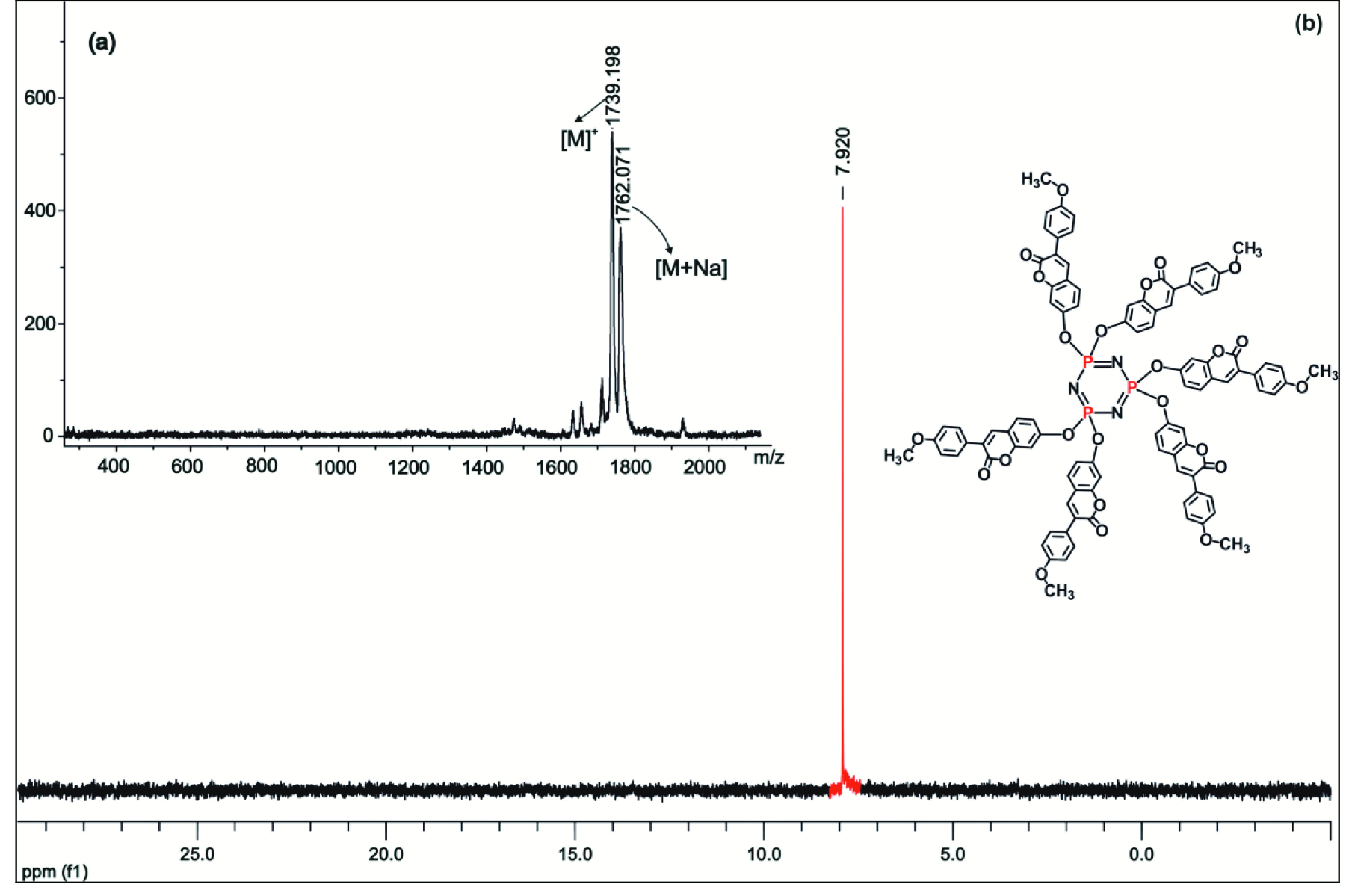
(a) MALDI-TOF spectrum of compound 5 and (b) the ^31^ P NMR spectrum in CDCl _3_ solution.

**Figure 2:**
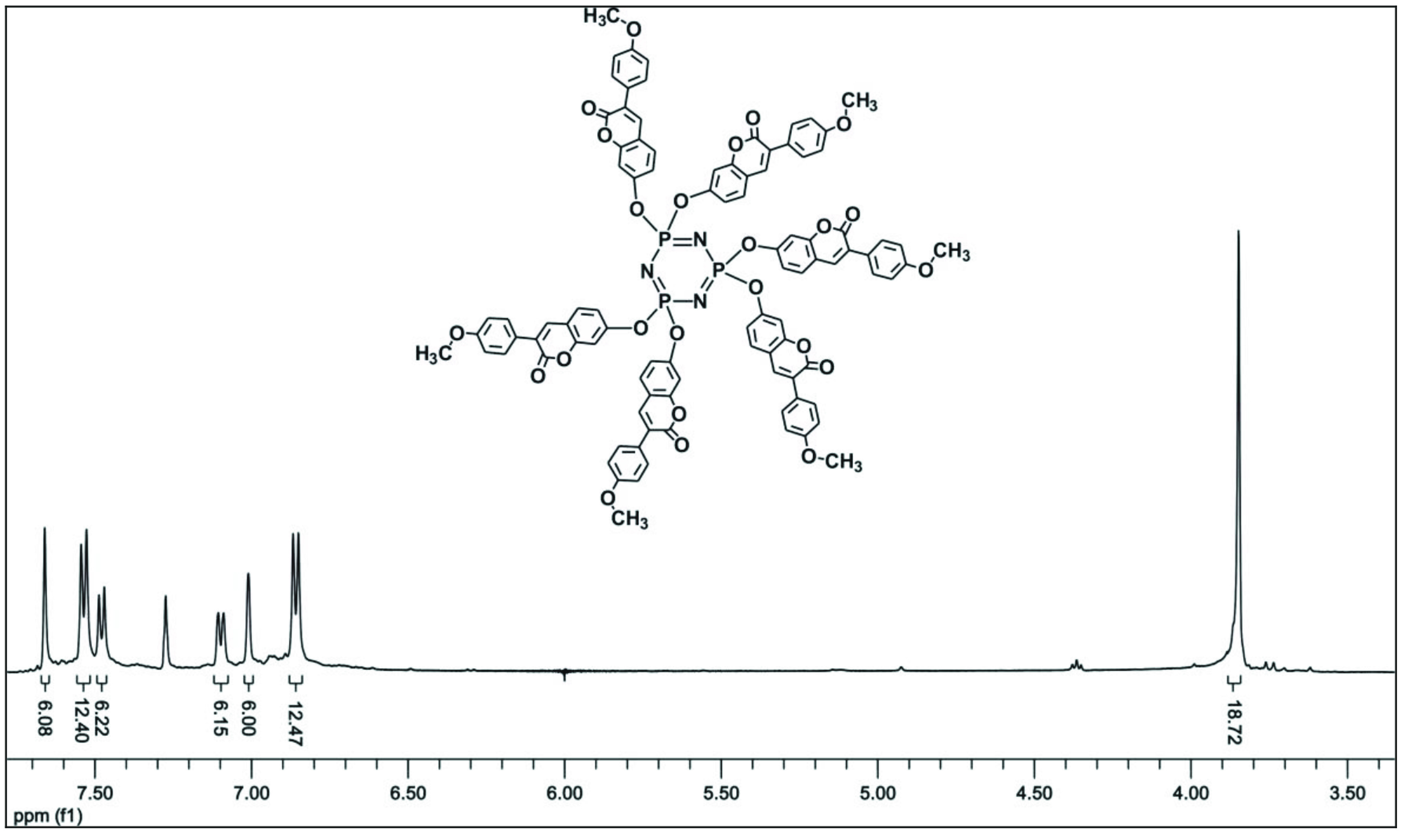
The ^1^ H NMR spectrum of compound 5 in CDCl _3_ solution.

Hexachlorocyclotriphosphazene (4) (0.5 g, 1.43 mmol) was dissolved with tetrahydrofuran in a 100-mL threenecked round-bottomed flask. After that, NaH (0.35 g, 8.58 mmol) dissolved with 5 mL of THF was added to the reaction mixture. Then 7-hydroxy-3-(4-methoxyphenyl)coumarin (1) (2.29 g, 8.58 mmol) dissolved with 10 mL of THF was added drop-wise for about 20 min and the reaction was heated with stirring at 80 ◦ C under an argon atmosphere for 4 days. After this time, the reaction mixture was filtered off with a G4 filter for removing formed NaCl salts. The solvent was completely removed by evaporation. Finally, the reaction mixture was purified by column chromatography using silica gel as column material and an n−hexane-THF (1:5) solvent mixture as an eluent. Yellowish pure product 5 was obtained (0.62 g, 0.35 mmol, 25%). Anal. Calc. for C _96_ H _66_ N _3_ O _24_ P _3_ , MALDI-TOF (m/z) calc. 1738.50, found: 1739.198 [M+H] ^+^ , 1762.071 [M+Na] ^+^ (Figure 1a). ^31^ P NMR (proton decoupled) (202 MHz, CDCl _3_ ) δP = 7.92 (s, 3P) ppm (Figure 1b). ^1^ H NMR (500 MHz; CDCl _3_ ) : δH = 7.66 (s, Ar-CH, 6H); 7.53 (d, Ar-CH, 12H, ^3^ J _H−H_ = 8.38); 7.47 (d, Ar-CH, 6H, ^3^ J _H−H_ = 8.41); 7.09 (d, Ar-CH, 6H, ^3^ J _H−H_ = 8.41); 7.01 (s, Ar-CH, 6H); 6.85 (d, Ar-CH, 12H, ^3^ J _H−H_ = 8.38); 3.84 (s, -OCH _3_ , 18H) ppm (Figure 2). FT-IR (cm ^-1^ ) : 3100–2800 (C-H Aromatic/Aliphatic); 1728 (C=O); 1610, 1575 (C=C), 1247 (C-O); 1146, 1121 (P=N); 990 (P-O).

#### 2.1.2. Synthesis of compound 6

Hexachlorocyclotriphosphazene (4) (0.5 g, 1.43 mmol) was dissolved with tetrahydrofuran in a 100-mL threenecked round-bottomed flask. After that, NaH (0.35 g, 8.58 mmol) dissolved with 5 mL of THF was added to the reaction mixture. Then 7-hydroxy-3-(3,4-dimethoxyphenyl)coumarin (2) (2.55 g, 8.58 mmol) dissolved with 10 mL of THF was added drop-wise for about 20 min and the reaction was heated with stirring at 80 ◦ C under an argon atmosphere for 4 days. After this time, the reaction mixture was filtered off with a G4 filter for removing formed NaCl salts. The solvent was completely removed by evaporation. Finally, the reaction mixture was purified by column chromatography using silica gel as column material and an n−hexane-THF (1:5) solvent mixture as an eluent. Yellowish pure product 6 was obtained (0.77 g, 0.40 mmol, 28%). Anal. Calc. for C _102_ H _78_ N _3_ O _3_ 0P _3_ , MALDI-TOF (m/z) calc. 1918.66, found: 1919.099 [M+H] ^+^ (Figure S1). ^31^ P NMR (proton decoupled) (202 MHz, CDCl _3_ ) δP = 7.99 (s, 3P) ppm (Figure S2). ^1^ H NMR (500 MHz; CDCl _3_ ) : ppm: δH = 8.06 (s, Ar-CH, 6H); 7.56 (d, Ar-CH, 6H, ^3^ J _H−H_ = 8.52); 7.28–7.24 (m, Ar-CH, 6H+6H); 6.98 (d, Ar-CH, 6H, ^3^ J _H−H_ = 8.35); 6.79 (d, Ar-CH, 6H, ^3^ J _H−H_ = 8.52); 6.73 (s, Ar-CH, 6H); 3.77 (s, -OCH _3_ , 18H); 3.76 (s, -OCH _3_ , 18H) ppm (Figure S3). FT-IR (cm ^-1^ ) : 3080–2850 (C-H aromatic/aliphatic); 1724 (C=O); 1610, 1514 (C=C); 1256 (C-O); 1144, 1119 (P=N); 997 (P-O).

#### 2.1.3. Synthesis of compound 7

Hexachlorocyclotriphosphazene (4) (0.5 g, 1.43 mmol) was dissolved with tetrahydrofuran in a 100-mL threenecked round-bottomed flask. After that, NaH (0.35 g, 8.58 mmol) dissolved with 5 mL of THF was added to the reaction mixture. Then 7-hydroxy-3-(3,4,5-trimethoxyphenyl)coumarin (3) (2.81 g, 8.58 mmol) dissolved with 10 mL of THF was added drop-wise for about 20 min and the reaction was heated with stirring at 80 ◦ C under an argon atmosphere for 4 days. After this time, the reaction mixture was filtered off with a G4 filter for removing formed NaCl salts. The solvent was completely removed by evaporation. Finally, the reaction mixture was purified by column chromatography using silica gel as column material and an n−hexane-THF (1:5) solvent mixture as an eluent. Then yellowish pure product 7 was obtained (0.63 g, 0.30 mmol, 21%). Anal. Calc. for C _108_ H _90_ N _3_ O _36_ P _3_ , MALDI-TOF (m/z) calc. 2097.45, found: 2097.05[M]+, 2117.05[M+Na] ^+^ (Figure S4). ^31^ P NMR (proton decoupled) (202 MHz, CDCl _3_ ) δP = 7.82 (s, 3P) ppm (Figure S5). ^1^ H NMR (500 MHz; CDCl _3_ ) : ppm: δH = 7.78 (s, Ar-CH, 6H); 7.58 (d, Ar-CH, 6H, ^3^ J _H−H_ = 8.51); 7.17 (d, Ar-CH, 6H, ^3^ J _H−H_ = 8.51); 6.97 (s, Ar-CH, 6H); 6.88 (s, Ar-CH, 12H); 3.89 (s, -OCH _3_ , 18H); 3.84 (s, -OCH _3_ , 36H) ppm (Figure S6). FT-IR (cm ^-1^ ) : 3090-2845 (C-H aromatic/aliphatic); 1724 (C=O); 1610, 1514 (C=C); 1256 (C-O); 1119, 1122 (P=N); 981 (P-O).

## 3. Results and discussion

### 3.1. Syntheses and characterizations

7-Hydroxycoumarin derivatives substituted cyclotriphosphazenes (5–7) were synthesized and their fluorescence chemosensor properties were also reported. 7-Hydroxy-3-(4-methoxyphenyl)coumarin (1), 7-hydroxy-3-(3,4-dimethoxyphenyl)coumarin (2), and 7-hydroxy-3-(3,4,5-trimethoxyphenyl)coumarin (3) were synthesized according to the procedures given in the literature [27–29]. Compounds 1–3 and hexachlorocyclotriphosphazene (4) were separately reacted in a 6:1 molar ratio in the presence of NaH as a base in THF under an argon atmosphere at room temperature (Scheme). All the products (5–7) were purified by column chromatography and characterized by MALDI-TOF MS, FT-IR, ^1^ H NMR, ^31^ P NMR, and fluorescence spectroscopy. The MS, ^1^ H NMR, and ^31^ P NMR results for each new compound were compatible with the data given in Section 2. All of the proton decoupled ^31^ P NMR spectra of compounds 5–7 showed a sharp single peak due to the equivalent chemical environment of all the phosphorus nuclei, suggesting an A3 spin system. The ^31^ P NMR spectrum of compound 5 is depicted as an example in Figure 1b.

The ^1^ H NMR data also confirmed the structures of compounds 5–7. The aromatic protons for all compounds were observed between 8.06 and 6.73 ppm and some of them were distinguishable from each other. The NMR signals of the -OCH _3_ groups were observed as a singlet or two singlets between 3.76 and 3.89 ppm. The ^1^ H NMR spectrum of compound 5 is given as an example in Figure 2. FT-IR frequencies of various diagnostic bands for compounds 5–7 are given in Section 2. These spectra showed characteristic stretching bands for aromatic/aliphatic -CH vibrations between 3100 and 2845 cm ^-1^ . The vibrations bands assigned to the stretching of the P=N- bands were observed at frequencies in the range of 1100–1150 cm ^-1^ .

### 3.2. Electronic absorption and fluorescence behavior

**Figure 3:**
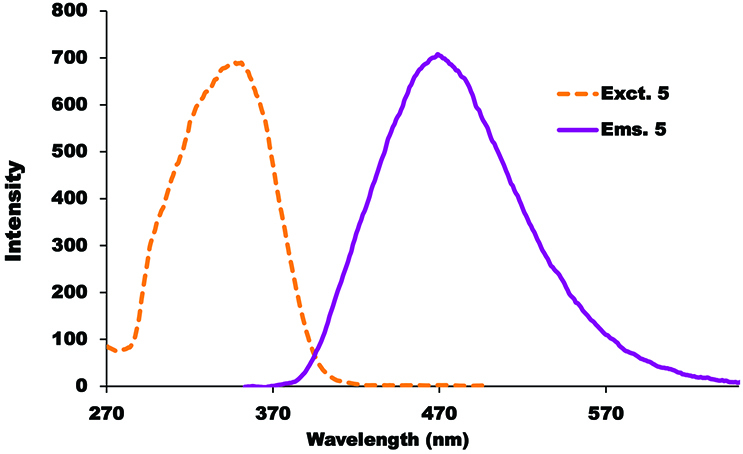
Excitation and emission spectra of compound 5 in THF/H _2_ O (20:1) solution. Excitation wavelength: 340 nm, C = 6 μM.

Absorption and emission properties of coumarin-based cyclotriphosphazene compounds were investigated in different solvents at room temperature (Figures S7–S9). The absorption spectra of the studied compounds (5–7) did not show any changes in the different studied solvents. The absorption bands observed at around 340 nm were attributed to the substituted coumarin groups because cyclotriphosphazenes are optically inert in the UV-Vis region [30]. In addition, the ground state absorption spectra of the compounds in THF/H _2_ O (20:1) were measured at different concentrations. From these spectra, the molar extinction coefficients of the compounds (5–7) were calculated according to the Beer–Lambert law (Figures S10–S12). The solvent effects on the fluorescence emission behavior of the studied compounds (5–7) were determined in different organic solvents as well as THF/H _2_ O (20:1) solution (Figures S13–S15). All studied coumarin substituted cyclotriphosphazenes (5–7) demonstrated similar fluorescence behavior (Figures 3, S16, and S17) and the maxima of the fluorescence emission peaks in THF/H _2_ O (20:1) solution are listed in the Table. The best fluorescence emission intensity with a large Stokes shift (158 nm) was observed for compound 7 in THF/H _2_ O (20:1) at 502 nm (Figure 3).

**Table:** Absorption, excitation, and emission spectral data for coumarin substituted cyclotriphosphazenes (5–7) THF/H _2_ O (20:1) solution.

Comp.	λmax (nm)	log ε	λEx (nm)	λEm (nm)	ΔStokes (nm)	τF (ns)
5	340	5.00	340	465	125	0.874 (20%), 3.876 (80%) (for 5)
						0.672 (21%), 3.839 (79%) (for 5+Fe)
6	351	4.88	352	498	146	0.956 (26%), 3.732 (73%) (for 6)
						1.035 (28%), 3.710 (72%)(for 6+Fe)
7	341	4.86	344	502	158	0.743 (85%), 2.865 (15%) (for 7)
						0.745 (87%), 3.175 (13%) (for 7+Fe)

**Figure 4:**
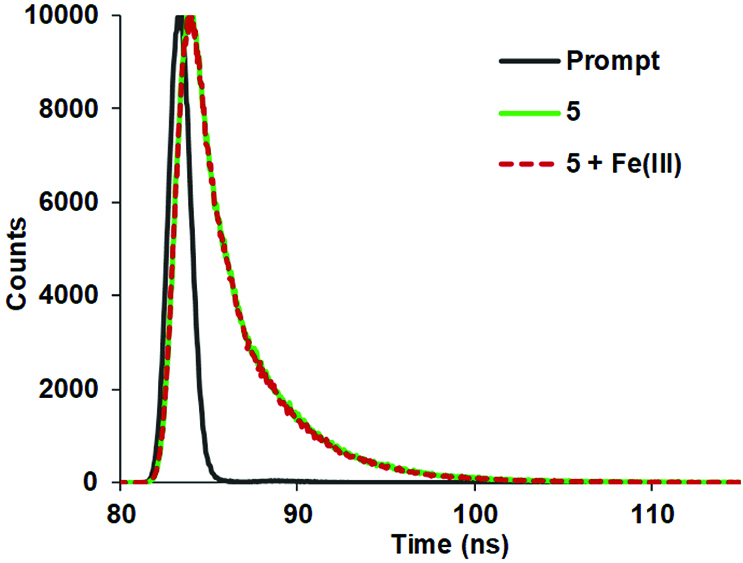
Fluorescence decay profiles of compound 5 in the absence and presence of Fe ^3+^ ions using laser excitation source of 390 nm.

The fluorescence lifetimes (τF ) of coumarin substituted cyclotriphosphazenes (5–7) were also measured using the time-correlated single-photon counting (TCSPC) technique (Figures 4, S18, and S19). The lifetimes were found to be biexponential and the τF values are given in the Table. Changes in the fluorescence lifetime values indicated the binding of Fe ^3+^ ions to the studied compounds (5–7).

### 3.3. Chemosensor properties

**Figure 5:**
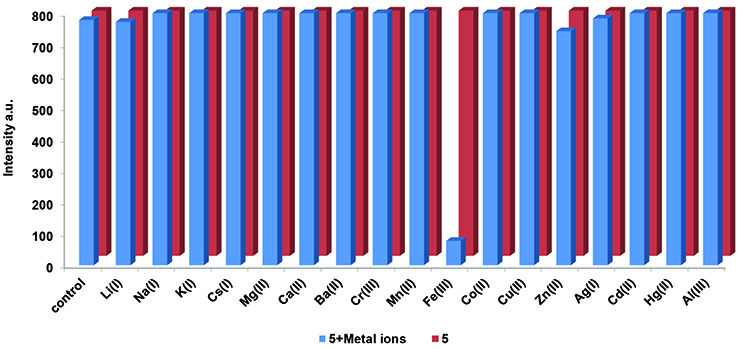
The fluorescence emission responses of cyclotriphosphazene compound 5 (6 μM) after addition of different metal ions.

The cation binding affinity of the newly synthesized coumarin substituted cyclotriphosphazenes (5–7) toward environmentally active metal ions was investigated using fluorescence spectroscopy (Figures 5, S20, and S21). Stock solutions of corresponding metal chlorides (nitrate derivative for Ag ^+^ ion) were prepared in water. Figure 5 shows that the addition of Li ^+^ , Na ^+^ , K ^+^ , Cs ^+^ , Mg ^2+^ , Ba ^2+^ , Ca ^2+^ , Cr ^3+^ , Mn ^2+^ , Co ^2+^ , Cu ^2+^ , Zn ^2+^ , Al ^3+^ , Ag ^+^ , Cd ^2+^ , and Hg ^2+^ caused virtually no change to the fluorescence spectra of the studied compounds at room temperature in THF/H _2_ O (20:1) solutions. Unlike the rest of the metal ions, Fe ^3+^ addition caused a dramatic decrease in the emission intensity of these compounds (5–7). The novel coumarin substituted cyclotriphosphazenes (5–7) showed ‘turn-off’ chemosensor properties towards the Fe ^3+^ ions in the solution.

**Figure 6:**
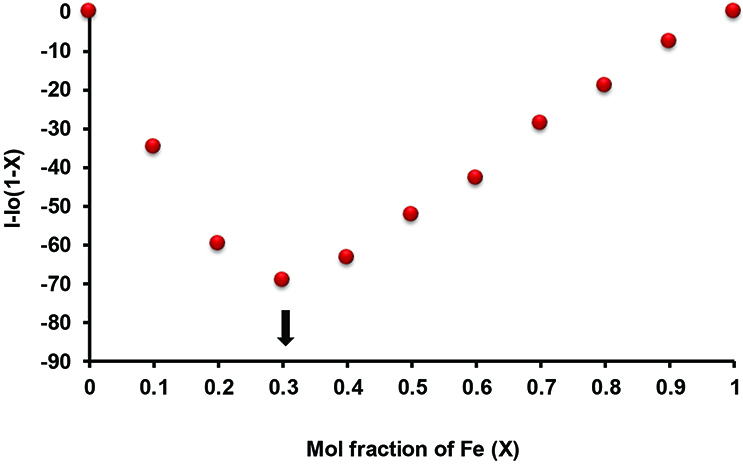
Job’s plot of 5-Fe ^3+^ complex in THF/H _2_ O (20:1) solution. Excitation wavelength = 340 nm.

The continuous variation method (Job’s plot) was used to determine the binding stoichiometry between the cyclotriphosphazene compounds (5–7) and Fe ^3+^ cation in THF/H _2_ O (20:1) solution. The resulting Job’s plot with a maximum mole fraction for the Fe ^3+^ cation was observed as 0.3, which indicates 2:1 (ligand:metal) binding stoichiometry for the complex formed between the chemosensor compound and Fe ^3+^ cation (Figures 6, S22B, and S23B).

**Figure 7:**
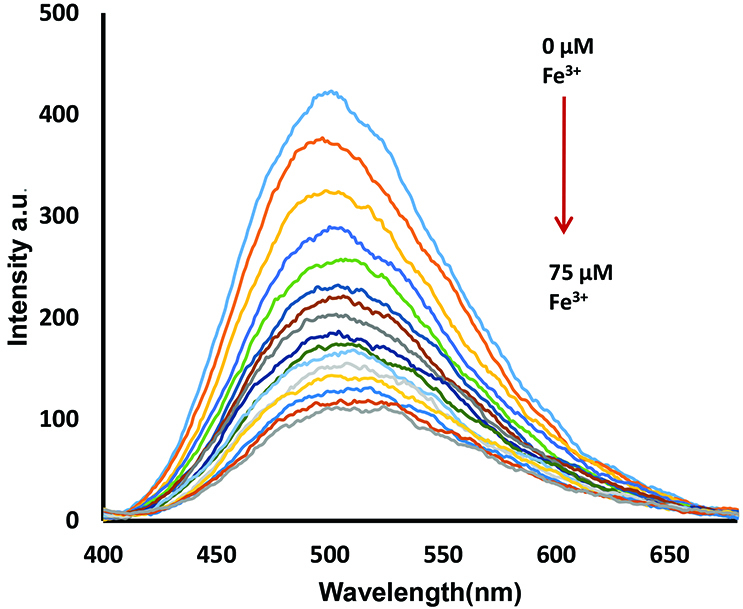
A) The fluorescence titration of compound 5 by addition of Fe ^3+^ cation [0–75 μM in THF/H _2_ O (20:1)] for determination of LOD. Excitation wavelength = 340 nm.

The limit of detection (LOD) (for compounds 5–7 with Fe ^3+^ ions were determined from fluorescence titration experiments using fluorescence spectroscopy (Figures 7, S22A, and S23A). This value was calculated using the 3σ/k equation (σ = standard deviation of the blank signal, k = slope of calibration curve) [31]. The LOD values were found as 4.32 μM, 2.19 μM, and 1.13 μM for compounds 5, 6, and 7, respectively, which allows detection in micromolar concentration ranges. These results showed that the novel coumarin substituted cyclotriphosphazene compounds (5–7) have high sensitivity towards Fe ^3+^ ions.

## 4. Conclusions

In this study, three novel 7-hydroxycoumarin substituted cyclotriphosphazene derivatives (5–7) were synthesized. All the synthesized novel compounds (5–7) were characterized by mass spectrometry, FT-IR, and ^1^ H and ^31^ P NMR spectroscopies. The electronic absorption and fluorescence behaviors of the synthesized cyclotriphosphazene derivatives were also studied by UV-Vis and fluorescence spectroscopy in THF/H _2_ O (20:1) solution. The novel studied cyclotriphosphazenes (5–7) were observed with absorption peaks at approximately 340 nm due to the optic properties of substituted coumarin. The fluorescence behaviors of the synthesized compounds were explored and intense emission bands were observed between 465 and 502 nm in THF/H _2_ O (20:1) solution. A significant decrease in the fluorescence emission intensities of the compounds (5–7) by the addition of the Fe ^3+^ cation solution was observed and this was evidence of the ‘turn-off’ chemosensor properties of these compounds against the Fe ^3+^ ions in the solution. These compounds could easily be used as chemosensors for Fe ^3+^ ions in solution because they showed low detection limits for Fe ^3+^ ions.

Supplementary Materials
