# Supplementary material for: Chemosensor properties of 7-hydroxycoumarin substituted cyclotriphosphazenes
Source: Turk J Chem. 2020 Feb 11;44(1):64–73. doi: 10.3906/kim-1908-51 (PMC7751823; doi:10.3906/kim-1908-51)
Supplement: Supplementary file 1 — Supplementary Materials [file turkjchem-44-64-sup001.pdf]

Supporting information

1. Experimental

1.1. Materials and methods

Hexachlorocyclotriphosphazene (98%), 3,4-methoxyphenylacetic acid (97%), 3,4-dimethoxyphenylacetic acid (97%), 3,4,5-trimethoxyphenylacetic acid (97%), and 2,4-dihydroxybenzaldehyde (98%) were obtained from Aldrich. Tetrahydrofuran (THF) (99.0%), dichloromethane (99.0%), and $n-$hexane (95.0%) were obtained from Merck. Sodium hydride (NaH) at 60% dispersion in mineral oil (Merck) was purified prior to use by washing with dry $n-$hexane followed by decantation. Silica gel 60 (230–400 mesh) for column chromatography was obtained from Merck. $CDCl_3$ for NMR spectroscopy was obtained from Goss Scientific. Positive ion and linear mode MALDI-MS of compounds was performed in dihydroxybenzoic acid as a MALDI matrix using a nitrogen laser accumulating 50 laser shots using a Bruker Microflex LT MALDI-TOF mass spectrometer. All reactions were monitored using thin-layer chromatography (TLC) on Merck silica gel plates (Merck, Kieselgel 60, 0.25 mm in thickness) with an F254 indicator. Column chromatography was performed on silica gel (Merck, Kieselgel 60, 230–400 mesh; for 3 g of crude mixture, 100 g of silica gel was used in a column of 3 cm in diameter and 60 cm in length). All reactions were carried out under an argon atmosphere. ^{1}H and ^{31}P NMR spectra were recorded in $CDCl_3$ solutions on a Varian INOVA 500 MHz spectrometer. Absorption spectra in the UV-Vis region were recorded with a Shimadzu 2101 UV-Vis spectrophotometer. Fluorescence excitation and emission spectra were recorded on a Varian Eclipse spectrofluorometer using 1-cm path length cuvettes at room temperature. The fluorescence lifetimes were obtained using a Horiba-Jobin-Yvon-SPEX Fluorolog 3-2iHR instrument with Fluoro Hub-B Single Photon Counting Controller at an excitation wavelength of 470 nm. Signal acquisition was performed using a TCSPC module (NanoLED-390 emitting 390 nm).

1.2. Chemosensor studies

Spectroscopic changes upon addition of metal salts to the ligand were recorded using a fluorescence spectrophotometer. All fluorescence emission spectral studies were performed in tetrahydrofuran/water (20:1) solutions (C = 6 μM) of the coumarin substituted cyclotriphosphazene compounds (**5**–**7**) at room temperature. The water solutions (C = 0.1 M) of the corresponding metal chlorides (nitrate derivative for Ag ion) were used as the source of metal ions. The UV-Vis spectra were routinely acquired at 25 °C in a 1-cm path length quartz cuvette with 2 mL of cyclotriphosphazene compounds (**5**–**7**) by the addition 0.1 mL of different metal solutions (Li^+, Na^+, K^+, Cs^+, Mg^{2+}, Ba^{2+}, Ca^{2+}, Cr^{3+}, Mn^{2+}, Fe^{3+}, Co^{2+}, Cu^{2+}, Zn^{2+}, Al^{3+}, Ag^+, Cd^{2+}, and Hg^{2+}). Titration experiments of cyclotriphosphazene compounds (**5**–**7**) in the presence of Fe^{3+} were carried out with freshly prepared stock solutions of cyclotriphosphazene compounds (**5**–**7**) in tetrahydrofuran/water (20:1) and Fe^{3+} solution in water using a fluorescence spectrophotometer.

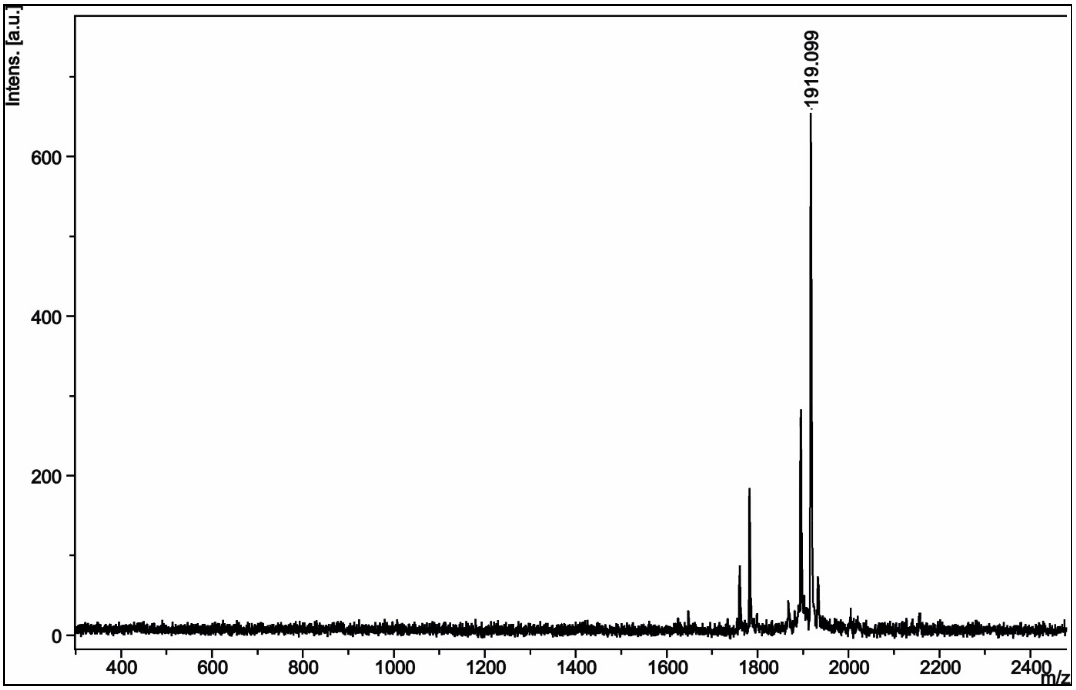

Figure S1. Mass spectrum of compound **6**.

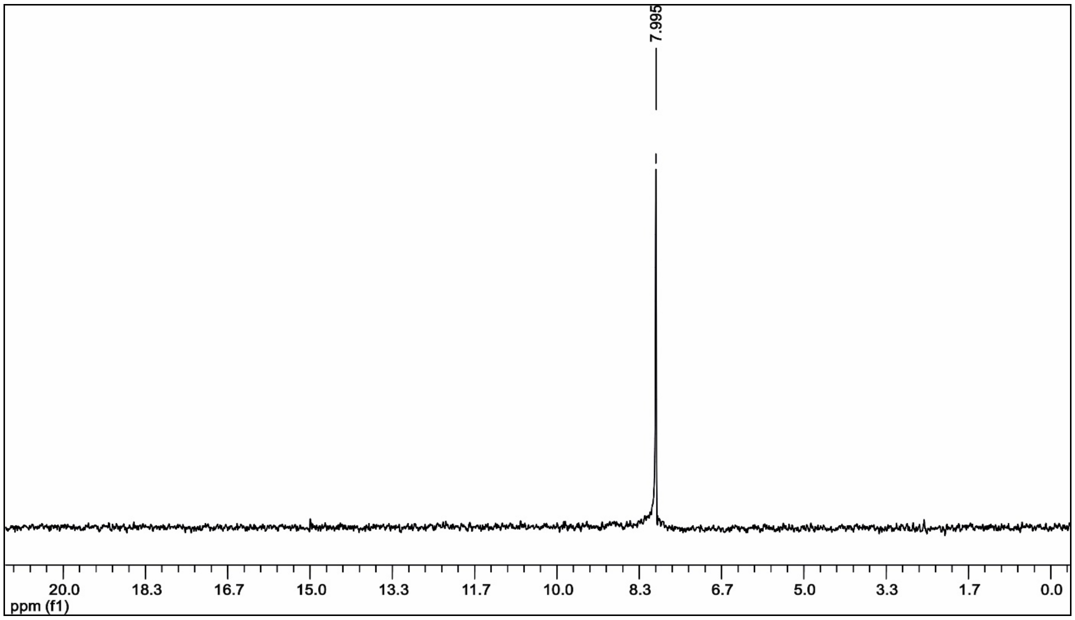

Figure S2. ^{31}P NMR spectrum of compound **6** in $CDCl_3$.

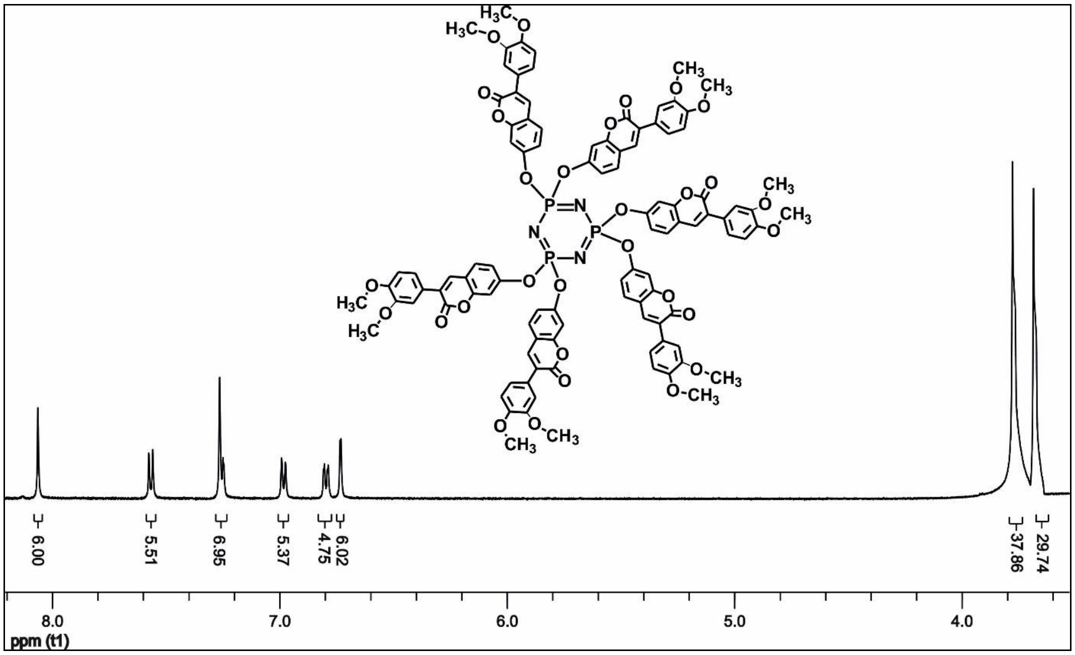

Figure S3. ^{1}H NMR spectrum of compound **6** in $CDCl_3$.

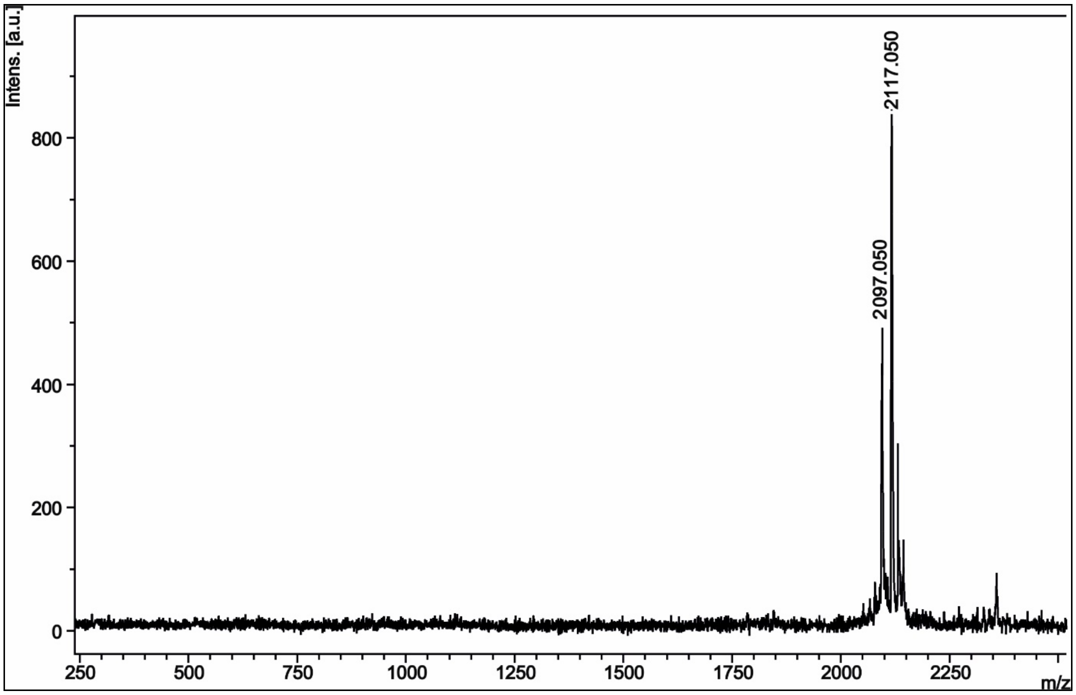

Figure S4. Mass spectrum of compound **7**.

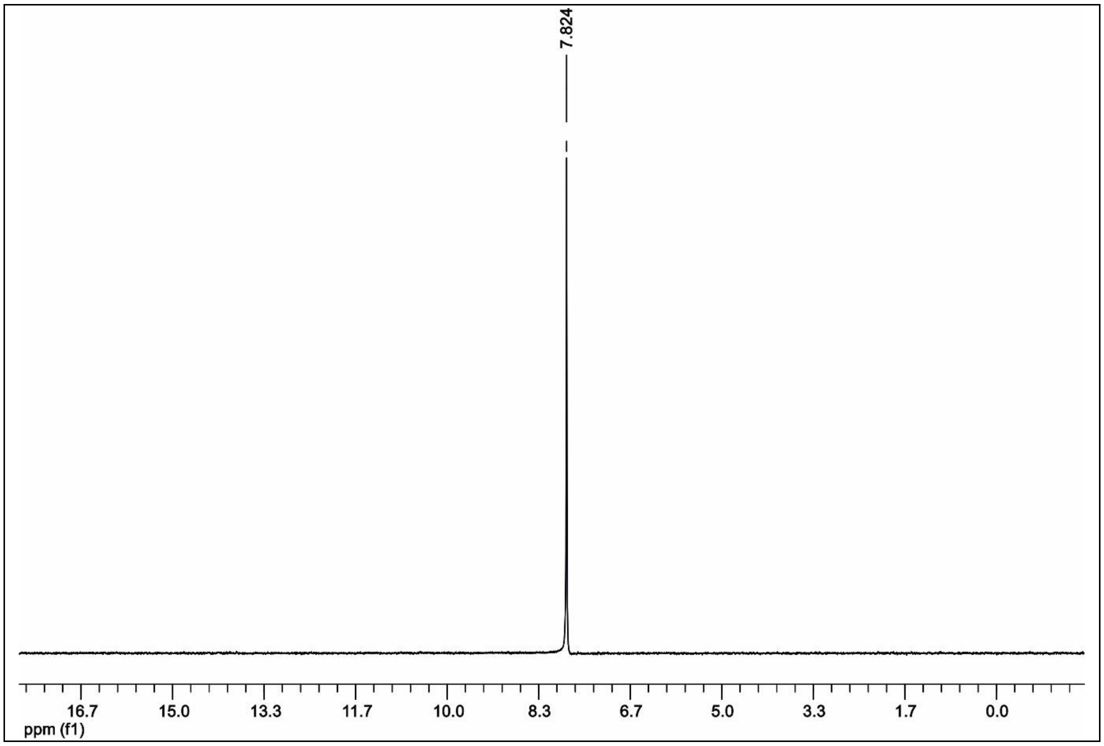

Figure S5. ^{31}P NMR spectrum of compound **7** in $CDCl_3$.

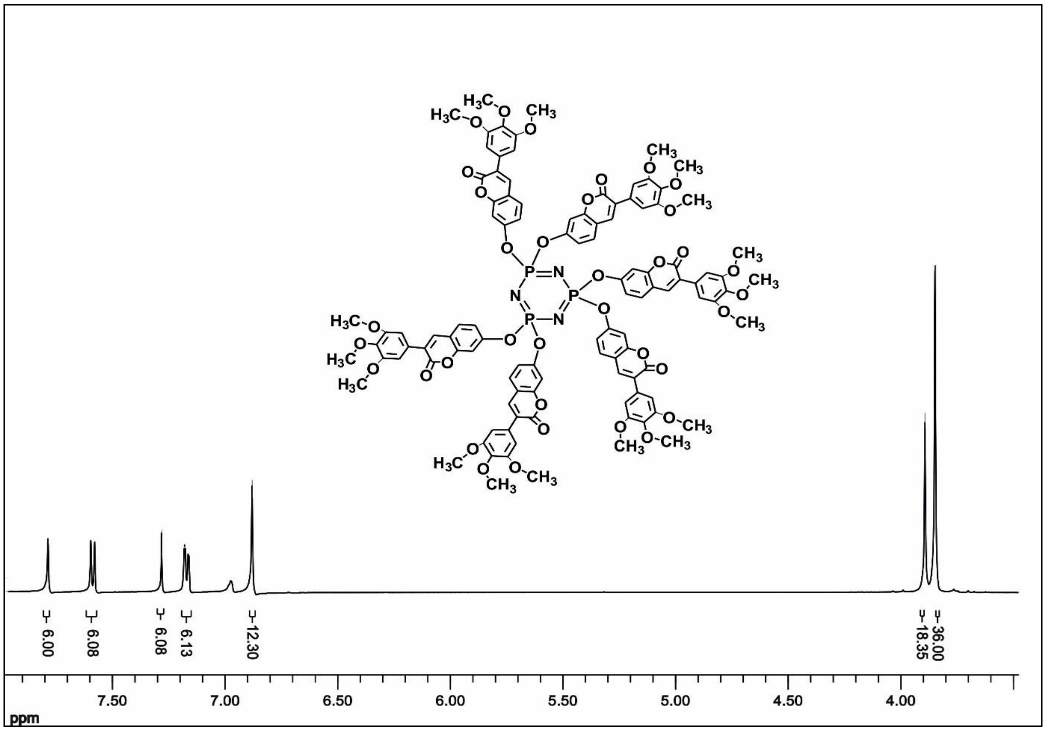

Figure S6. ^{1}H NMR spectrum of compound **7** in $CDCl_3$.

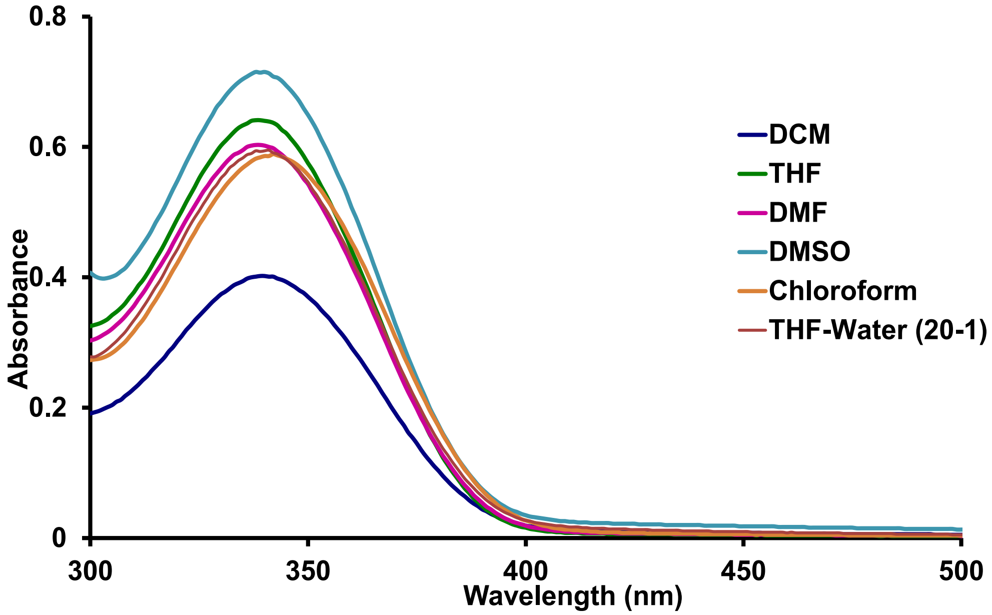

Figure S7. UV-Vis absorption spectra of compound **5** in different solutions.

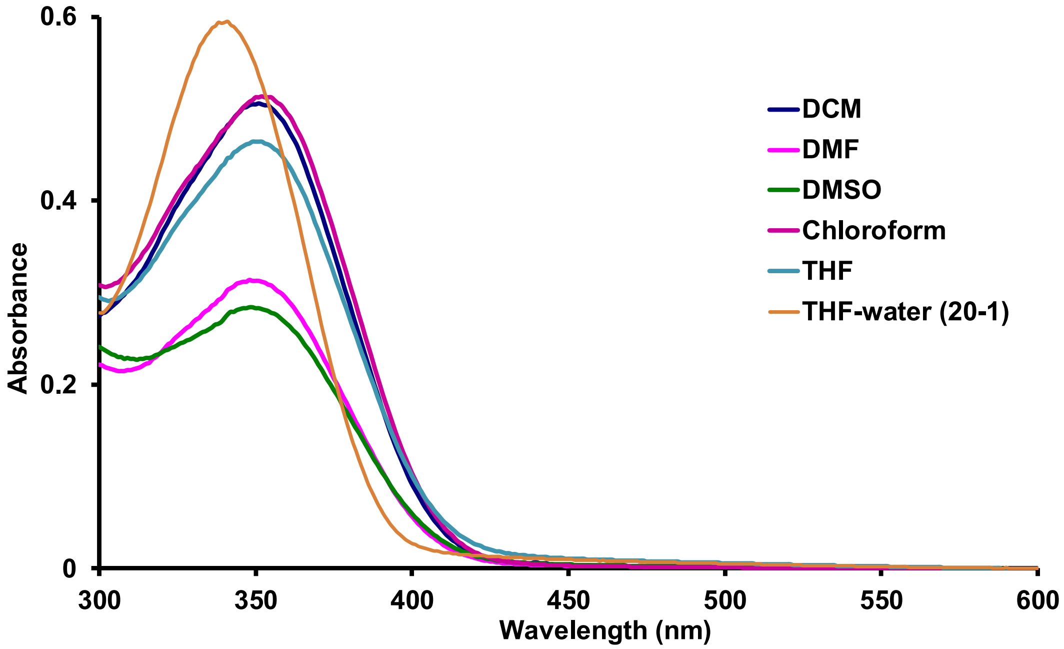

Figure S8. UV-Vis absorption spectra of compound **6** in different solutions.

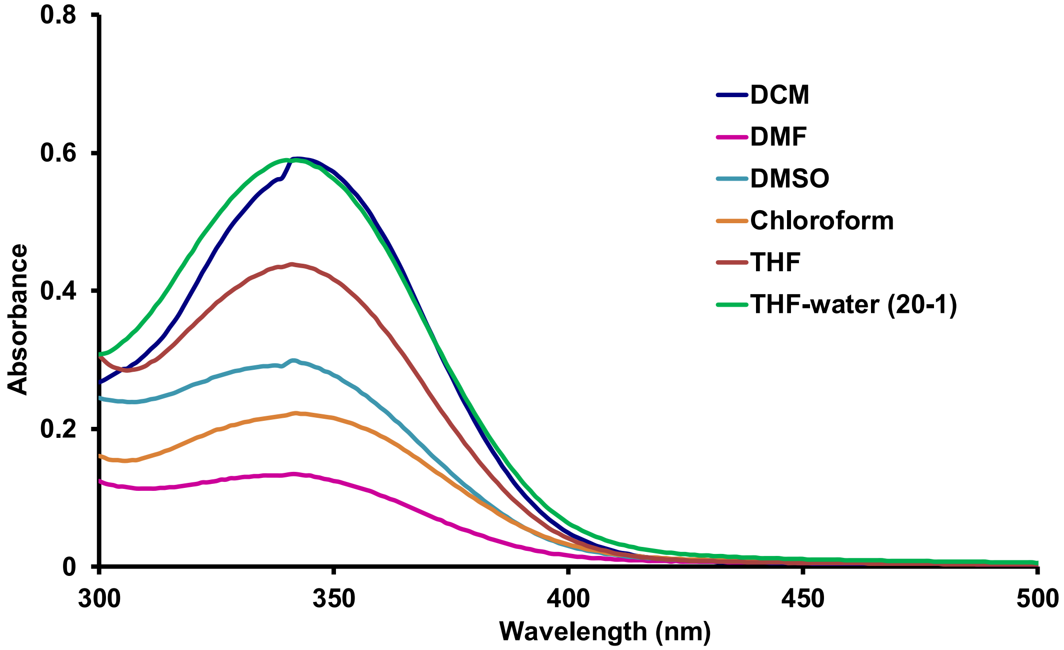

Figure S9. UV-Vis absorption spectra of compound **7** in different solutions.

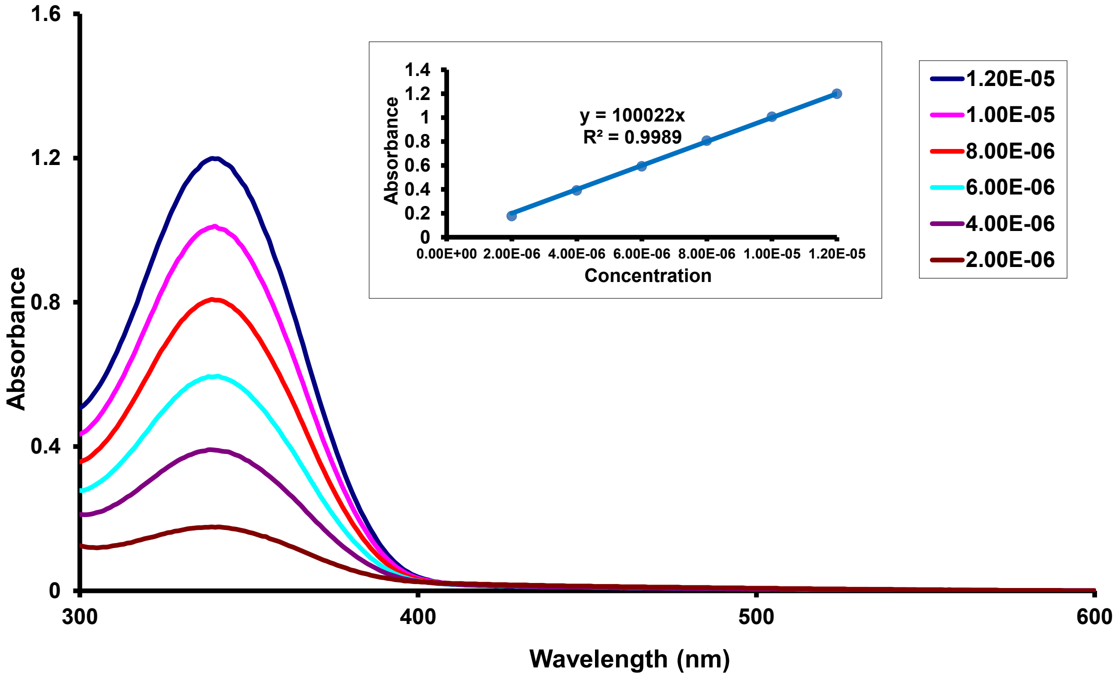

Figure S10. Molar extinction coefficient of compound **5** in 20-1 THF-water solution.

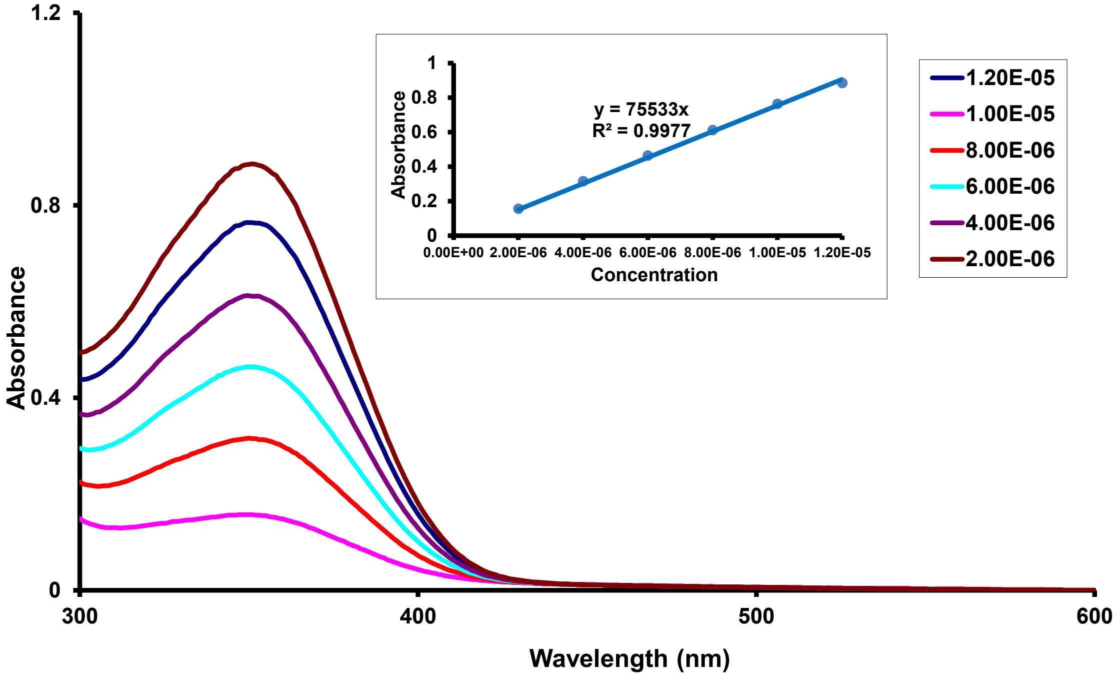

Figure S11. Molar extinction coefficient of compound **6** in 20-1 THF-water solution.

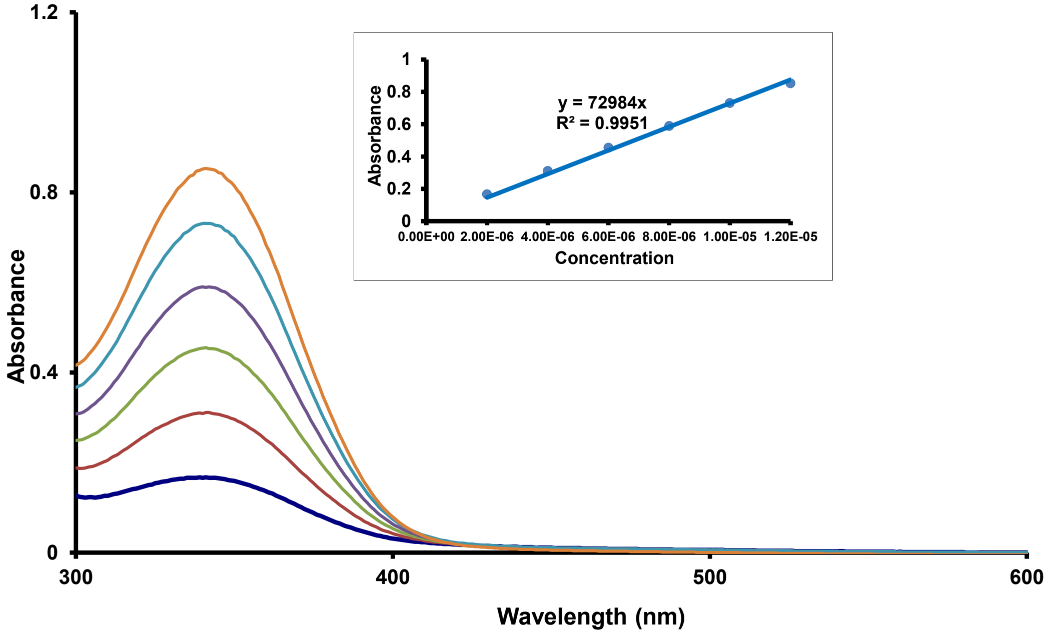

Figure S12. Molar extinction coefficient of compound **7** in 20-1 THF-water solution.

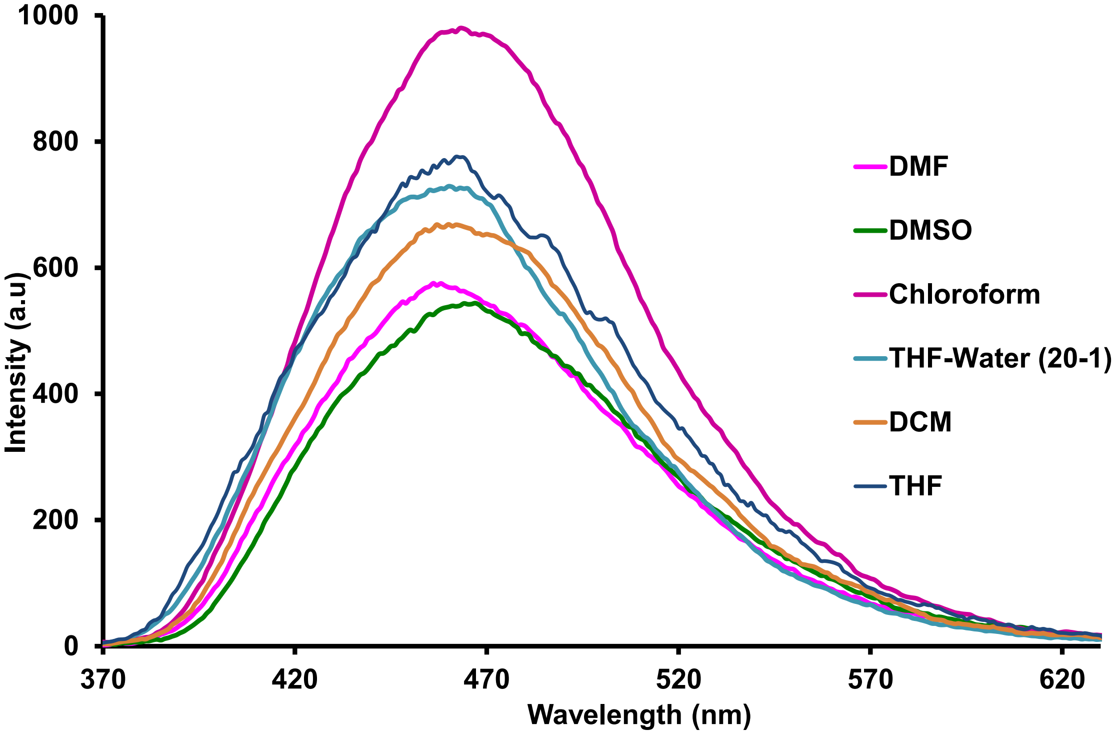

Figure S13. Fluorescence emission spectra of compound **5** in different solutions.

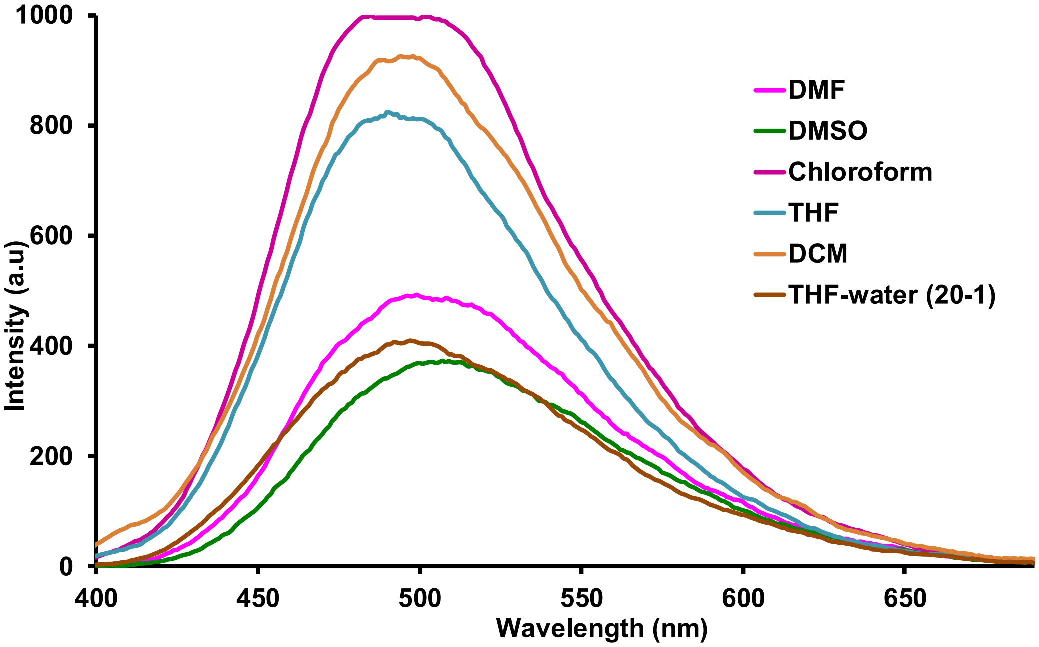

Figure S14. Fluorescence emission spectra of compound **6** in different solutions.

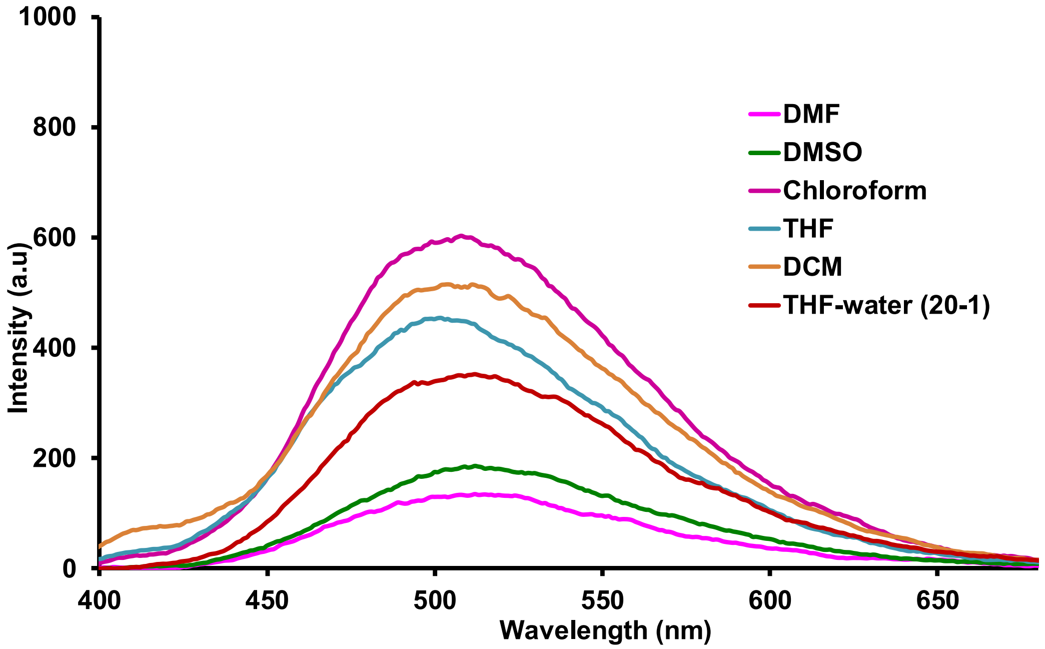

Figure S15. Fluorescence emission spectra of compound **7** in different solutions.

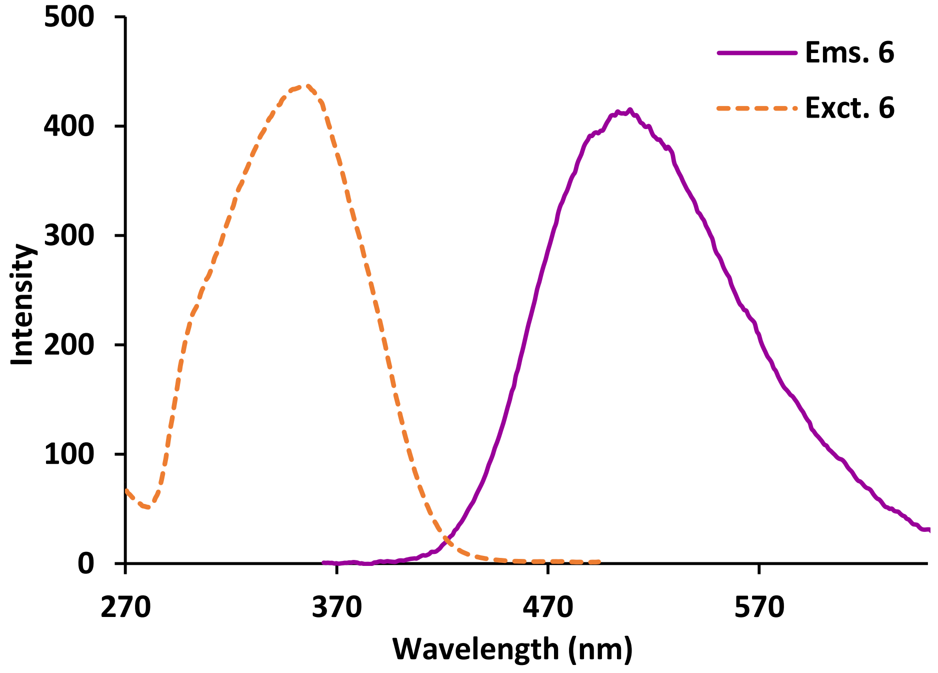

Figure S16. Excitation (dashed lines) and emission spectra (solid lines) of compound **6** in THF-H_2O (20/1).

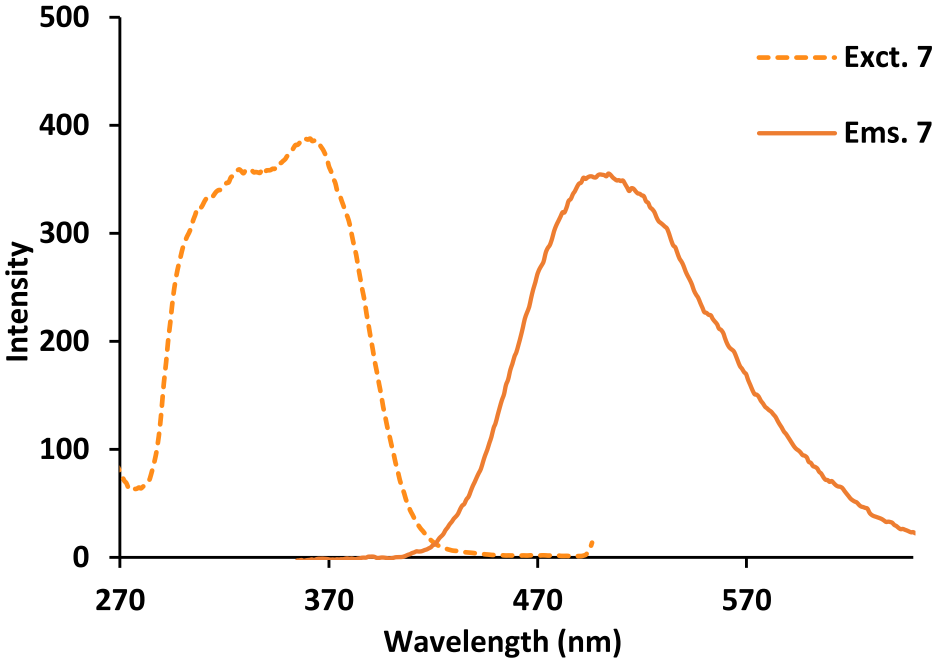

Figure S17. Excitation (dashed lines) and emission spectra (solid lines) of compound **7** in THF-H_2O (20/1).

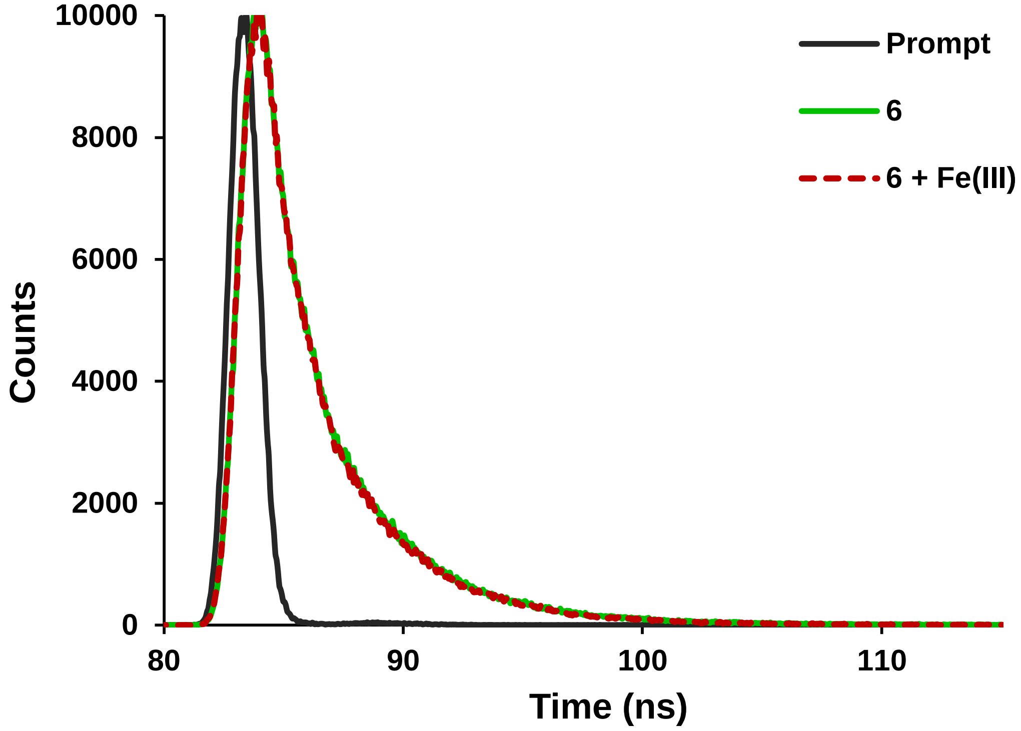

Figure S18. Fluorescence decay profiles of compound **6** in the absence and presence of Fe^{3+} ion using laser excitation source of 390 nm.

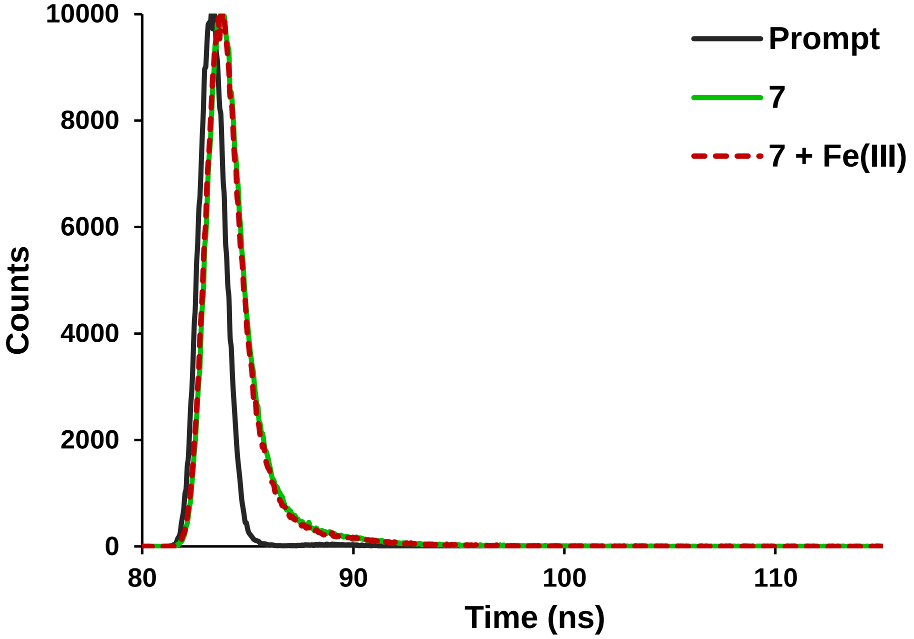

Figure S19. Fluorescence decay profiles of compound **7** in the absence and presence of Fe^{3+} ion using laser excitation source of 390 nm.

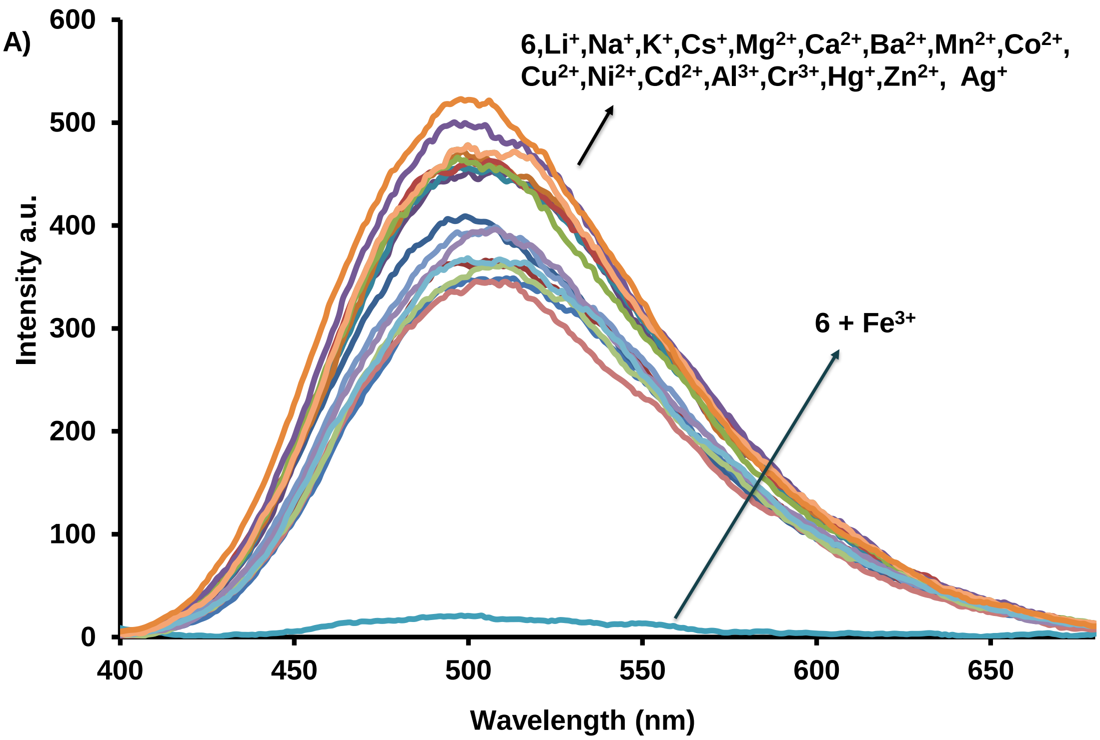

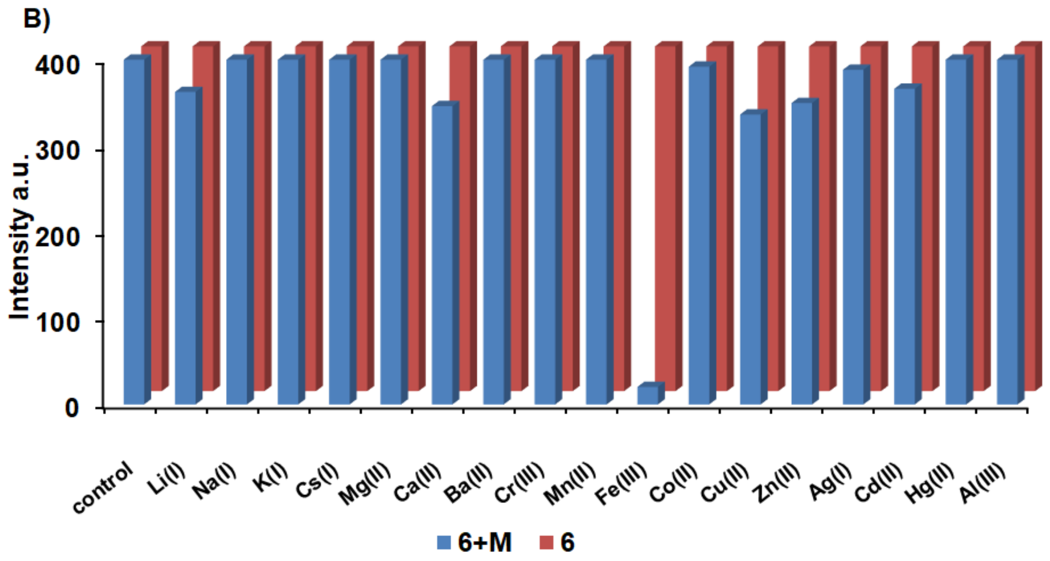

Figure S20. A) Fluorescence emission spectra and B) bar graph of compound **6** (6 μM) with the addition of metal ions.

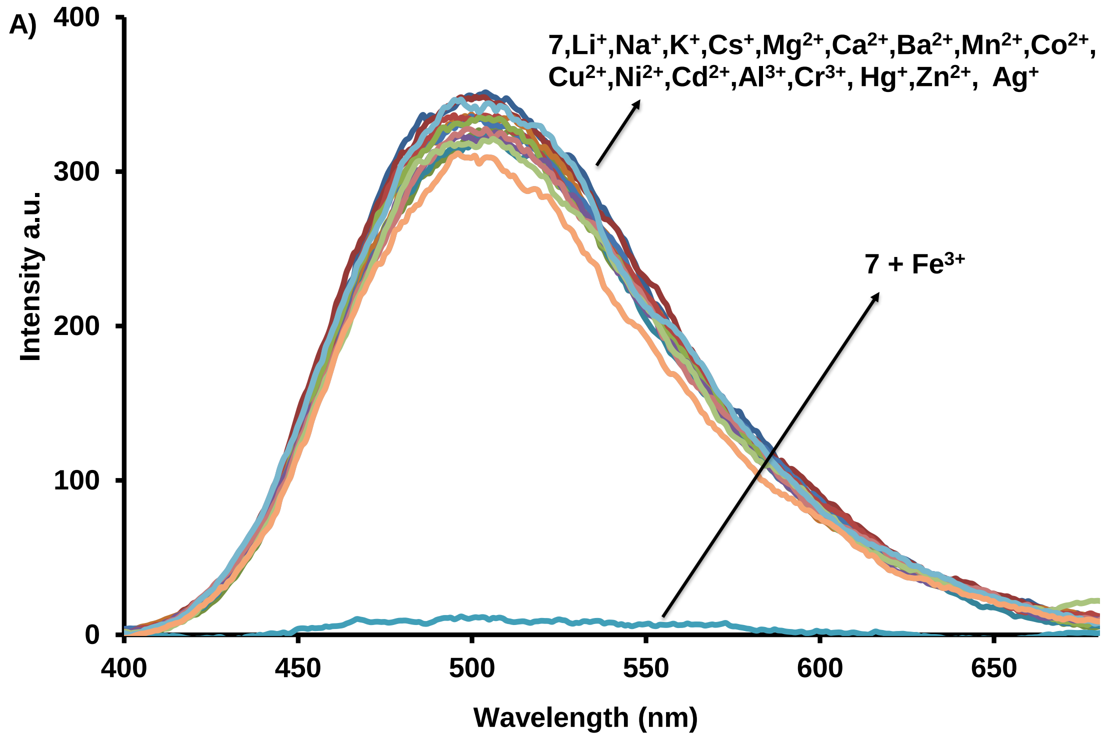

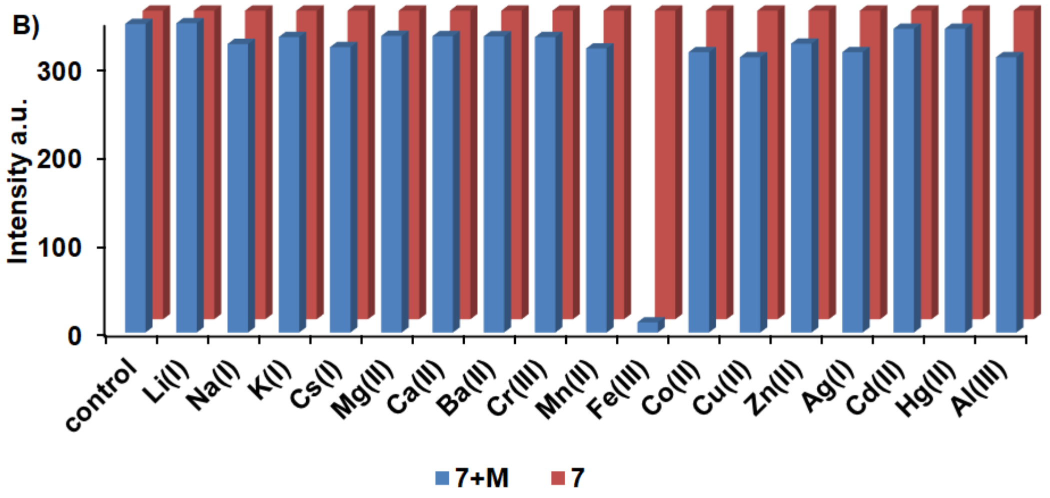

Figure S21. A) Fluorescence emission spectra and B) bar graph of compound **7** (6 μM) with the addition of metal ions.

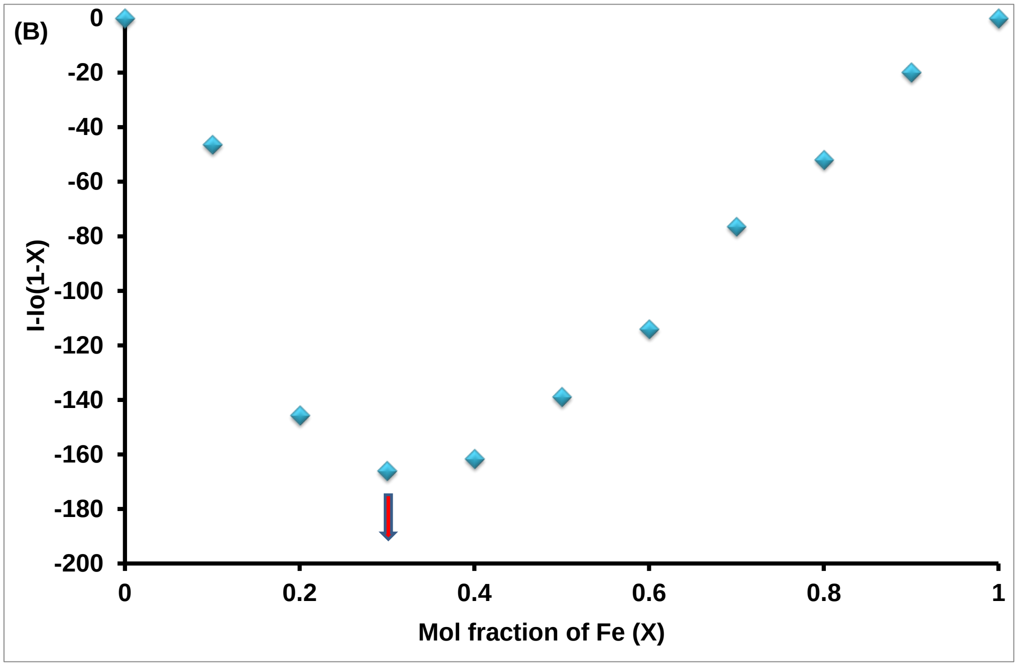

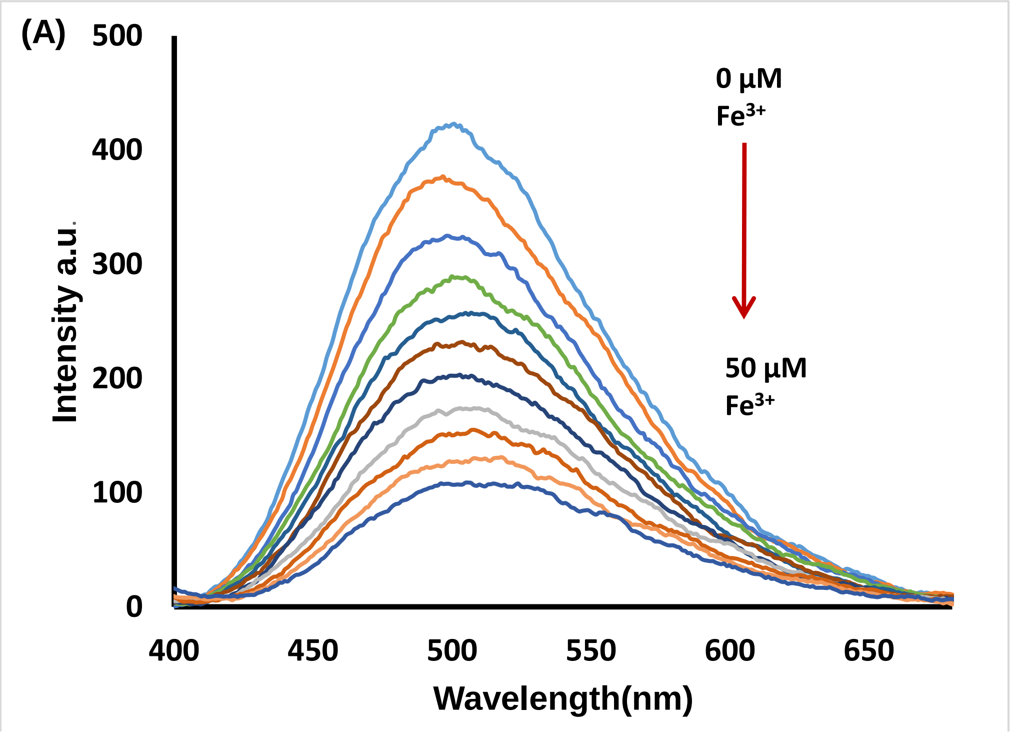

Figure S22. A) Job's graph and B) the fluorescence titration of compound **6** by addition of Fe^{3+} cation [0–50 µM in THF/water (20:1)] for determination of LOD (excitation wavelength = 340 nm).

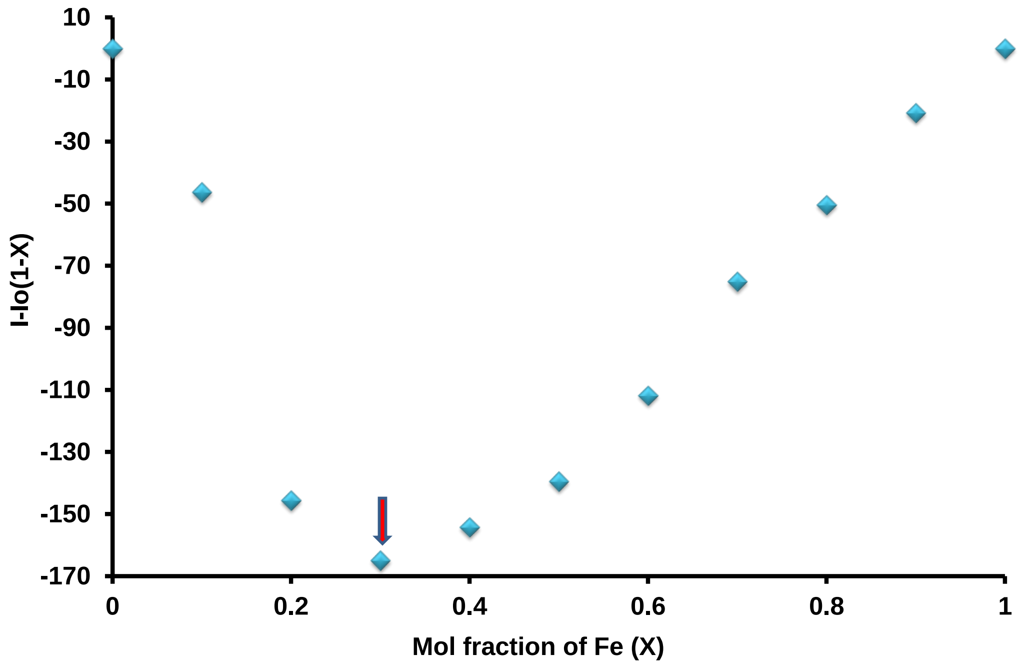

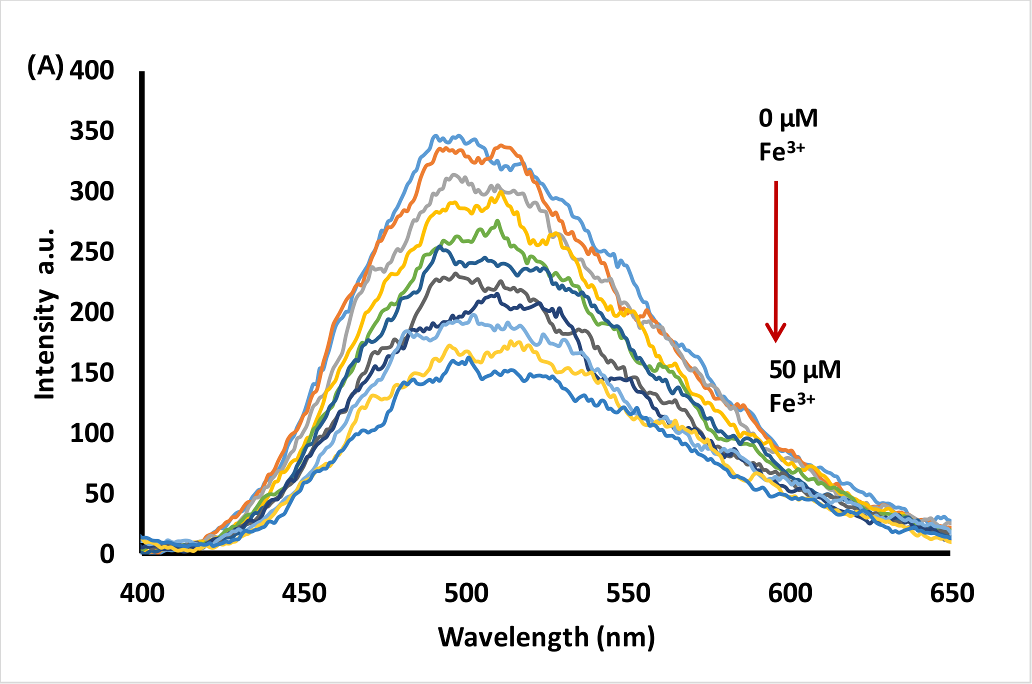

Figure S23. A) Job's graph and B) the fluorescence titration of compound **7** by addition of Fe^{3+} cation [0–50 μM in THF/water (20:1)] for determination of LOD (excitation wavelength = 340 nm).
